# Letter to the editor: “Validation of the Connecticut olfactory test (CCCRC) adapted to Brazil”

**DOI:** 10.1016/j.bjorl.2021.09.007

**Published:** 2021-11-05

**Authors:** Henrique de Paula Bedaque, Maria Luísa Nobre Medeiros e Silva Guimarães, Lidiane Maria de Brito Macedo Ferreira

**Affiliations:** Universidade Federal do Rio Grande do Norte (UFRN), Natal, RN, Brazil

Dear Editor,

I am writing regarding an article of great relevance to otorhinolaryngology published in this renowned journal, i.e., “Validation of the Connecticut olfactory test (CCCRC) adapted to Brazil”, by Fenólio et al.,^1^ which brought attention to the validation of an olfactory function assessment test. This is a low-cost test with great clinical relevance, especially regarding the identification of a large number of patients with olfactory dysfunction as one of the sequelae of SARS-CoV-2 infection, in addition to Parkinson’s disease and other diseases with olfactory involvement.

In view of this validation, the otorhinolaryngology division of the University Hospital Onofre Lopes of the Federal University of Rio Grande do Norte initiated its own production of the CCCRC test. We believe that some difficulties we experienced during the production process may positively contribute, so that other services will be able to produce this test in a safer and more standardized way.

Initially, it is important to emphasize that butanol (butyl alcohol) used at different dilutions in the test is a flammable, corrosive, and volatile substance.^2^ This means that, when obtaining a standard bottle of butanol (99%), it must be handled in a proper laboratory and diluted in a hood, with a three-month expiration date. Subsequently, after the vials are packaged in amber glasses, they become safer to be handled, although one must always be aware that the vial containing the highest concentration (Butanol 4%) is still flammable.^2^

Moreover, when presenting the butanol bottle to the patient, we noticed that some important information, such as the distance from the bottle to the nostril and the time during which it should be close to the nostril until the end of the step, were missing. There was no such standardization in the validation article^1^ and in the pioneer article of the CCCRC^3^ a plastic bottle, susceptible to compression, was used to force the escaping of gases, which we currently know is an inadequate way of packaging the substance.^2^

Therefore, our center team adopted, as a standard procedure, making three circular movements for each bottle before opening the cap with subsequent presentation to the patient for up to 5 s, during which the bottle mouthpiece will always be at the same level of the patient’s upper lip. Hence, we believe that these relevant information may help other services to carry out this important test in a more standardized way, allowing new studies and making it more homogeneous and reproducible.

## Conflicts of interest

The authors declare no conflicts of interest.
